# Target-site cefiderocol pharmacokinetics in soft tissues of healthy volunteers

**DOI:** 10.1093/jac/dkae359

**Published:** 2024-10-07

**Authors:** Maria Sanz-Codina, Wisse van Os, Anh Duc Pham, Anselm Jorda, Michael Wölf-Duchek, Felix Bergmann, Edith Lackner, Constantin Lier, J G Coen van Hasselt, Iris K Minichmayr, Christoph Dorn, Markus Zeitlinger, Valentin al Jalali

**Affiliations:** Department of Clinical Pharmacology, Medical University of Vienna, Vienna, Austria; Department of Clinical Pharmacology, Medical University of Vienna, Vienna, Austria; Leiden Academic Centre for Drug Research, Leiden University, Leiden, The Netherlands; Department of Clinical Pharmacology, Medical University of Vienna, Vienna, Austria; Department of Clinical Pharmacology, Medical University of Vienna, Vienna, Austria; Department of Biomedical Imaging and Image-guided Therapy, Medical University of Vienna, Vienna, Austria; Department of Clinical Pharmacology, Medical University of Vienna, Vienna, Austria; Department of Plastic, Reconstructive and Aesthetic Surgery, Medical University of Vienna, Vienna, Austria; Department of Clinical Pharmacology, Medical University of Vienna, Vienna, Austria; Institute of Pharmacy, University of Regensburg, Regensburg, Germany; Leiden Academic Centre for Drug Research, Leiden University, Leiden, The Netherlands; Department of Clinical Pharmacology, Medical University of Vienna, Vienna, Austria; Institute of Pharmacy, University of Regensburg, Regensburg, Germany; Department of Clinical Pharmacology, Medical University of Vienna, Vienna, Austria; Department of Clinical Pharmacology, Medical University of Vienna, Vienna, Austria

## Abstract

**Background:**

Cefiderocol may potentially be used to treat skin and soft tissue infections (SSTIs). However, the pharmacokinetics of cefiderocol in human soft tissues have not yet been determined. The objective of the present PK study was to investigate whether target-site concentrations of cefiderocol are sufficiently high for the treatment of SSTIs.

**Methods:**

In this pharmacokinetic study, a single intravenous dose of 2 g cefiderocol was administered to eight healthy male volunteers. Drug concentrations were determined in plasma, muscle and subcutis over 8 h. Free plasma concentrations were calculated using the plasma protein binding determined with ultrafiltration. Free tissue concentrations were obtained using microdialysis. Penetration ratios were calculated as AUC_0-8h_free_tissue_/AUC_0-8h_free_plasma_. A population pharmacokinetic model was developed, and the probability of target attainment (PTA) was determined using Monte Carlo simulations.

**Results:**

Cefiderocol showed good tissue penetration, with mean penetration ratios ± standard deviation of 0.99 ± 0.33 and 0.92 ± 0.30 for subcutis and muscle, respectively. Cefiderocol pharmacokinetics in plasma were best described with a two-compartment model, and tissue concentrations were described by scaling the tissue concentrations to concentrations in the peripheral compartment of the plasma model. For a thrice-daily regimen with 2 g doses intravenously infused over 3 h, PTA was ≥90% for MIC values up to 4 mg/L, both based on free plasma and soft tissue pharmacokinetics.

**Conclusions:**

This study indicates that a dose of 2 g cefiderocol achieves concentrations in plasma considered sufficient for treating relevant bacterial species. Assuming a comparable PK/PD target for soft tissues, sufficiently high concentrations would also be achieved in soft tissues.

## Introduction

The increasing multidrug resistance (MDR) of Gram-negative bacteria (GNB) presents a significant challenge to worldwide health.^1^ Skin and soft tissue infections (SSTIs) caused by MDR-GNB are on the rise, especially in patients with underlying immunodeficiencies, diabetes mellitus and burn or trauma injuries.^2–4^ New effective and safe antibiotics against MDR-GNB SSTIs are therefore urgently needed.

Cefiderocol is a new siderophore cephalosporin that might address this unmet need. In contrast to many older cephalosporin antibiotics, cefiderocol demonstrates stability to serine- and metallo-beta-lactamases.^5^ However, it has minimal activity against Gram-positive or anaerobic bacteria due to intrinsic resistance.^6^

The efficacy and safety of cefiderocol have been investigated in patients with complicated urinary tract infections (cUTIs), hospital-acquired bacterial pneumonia and ventilator-associated bacterial pneumonia (HABP/VABP) who were at risk of being infected by MDR or carbapenem-resistant GNB.^7,8^ Cefiderocol has been approved in Europe for the treatment of infections due to aerobic GNB in adults with limited treatment options^9^ and in the USA for the treatment of HABP/VABP and cUTIs caused by susceptible Gram-negative microorganisms.^10^

Cefiderocol is primarily eliminated through the kidneys, with minimal hepatic metabolism.^11^ Following a 2000 mg intravenous dose over 1 h, it achieved a maximum plasma concentration (C_max_) of approximately 156 mg/L.^12^ The volume of distribution (Vd) is around 18 L, and its elimination half-life (t_1/2_) is 2–3 h, necessitating adjustments based on renal function to maintain therapeutic levels.^12,13^

Cefiderocol has been proposed for the treatment of SSTIs caused by MDR-GNB, due to its good activity profile.^2^ However, to date, no data exist regarding cefiderocol penetration into soft tissues. The aim of the present study was to investigate the pharmacokinetics (PK) of cefiderocol in plasma, skeletal muscle and subcutaneous adipose tissue of healthy volunteers and determine probability of target attainment (PTA), thereby enhancing our understanding of the drug exposure at potentially relevant infection sites.

## Methods

### Ethics

The Ethics Committee of the Medical University of Vienna (EC number: 2465/2020) and the Austrian Agency for Health and Food Safety both gave their approval before the start of the study. EudraCT number 2020-005714-17 was issued to the study. Participants provided oral and written informed consent before inclusion in the study. The International Conference on Harmonization-Good Clinical Practice (ICH-GCP) recommendations and the Declaration of Helsinki were followed during subject-related study procedures at the Department of Clinical Pharmacology at the Medical University of Vienna, Austria.

### *In vitro* microdialysis experiments

*In vitro* microdialysis (MD) experiments were performed to determine the feasibility of *in vivo* MD with cefiderocol. Specifically, *in vitro* MD allows to evaluate whether microdialysis works with the test compound by evaluating the magnitude of recovery rates (RR), test if RR are constant over time and test if RR are constant at different drug concentrations. Finally, the *in vitro* MD experiments serve to assess if recovery is equal in both directions of the membrane. One direction is from the immersion solution through the membrane into the collecting vial and called forward dialysis. The other direction is from the perfusion solution of the pump through the membrane into the immersion solution and called retrodialysis dialysis. Equal recovery in both directions is an important requisite for calibrating the MD probes with the retrodialysis technique in the *in vivo* experiments. The details of *in vitro* MD experiments have been reported previously in detail by MacVane *et al.*^14^ and graphically summarized in Figure S1 (available as Supplementary data at *JAC* Online).

The experiments were performed in triplicates in a shaking water bath at 37°C. Three MD catheters (type ‘63’ MD catheter, M Dialysis AB, Stockholm, Sweden) with membranes of a molecular weight cut-off of 20 kDa and a membrane length of 10 mm were used. MD Catheters were connected with precision pumps (107 MD pump; M Dialysis AB, Stockholm, Sweden). The immersion solutions were placed in 10 mL glass vials, and plastic vials were used for the collection of the microdialysate.

For the forward dialysis experiments, three MD probes were placed separately in glass vials containing the cefiderocol solution and subsequently perfused with 0.9% saline solution at a flow rate of 2 µL/min. After an equilibration period of at least 60 min, four consecutive microdialysate samples over two 30 min intervals (0–30 min, 30–60 min) and two 60 min intervals (60–120 min, 120–180 min) were collected from each of the three probes before placing the probes in the next vial with higher cefiderocol concentrations. These procedures were performed using 3, 15 and 75 μg/mL cefiderocol solutions. For the retrodialysis experiments, the cefiderocol solutions were used as perfusion solutions and saline (0.9%) was used as the immersion solution. Sampling was performed as described for forward dialysis. Immersion and perfusion solution aliquots were collected before and at the end of each sampling period.

Forward dialysis recovery was calculated according to the following equation:


$$\mathrm{Recovery}\;(\text{\%})=100\times \frac{\mathrm{concentration}\;\mathrm{in}\;\mathrm{MD}\;\mathrm{vial}}{\mathrm{concentration}\;\mathrm{in}\;\mathrm{immersion}\;\mathrm{solution}}.$$

Retrodialysis recovery was calculated according to the following equation:


$$\mathrm{Recovery}\;(\text{\%})=100-\left(100\times \frac{\mathrm{concentration}\;\;\mathrm{in}\;\;\mathrm{MD}\;\;\mathrm{vial}}{\mathrm{concentration}\;\;\mathrm{in}\;\;\mathrm{perfusion}\;\;\mathrm{solution}}\right).$$

### Study population and *in vivo* procedures

Before enrolment in the study, eight volunteers underwent a screening examination that included a physical examination, an electrocardiogram, blood pressure measurement and blood sampling (haematology, chemistry, coagulation and virology testing). Major inclusion criteria were: healthy male subjects aged 18–55 years; body mass index within a range of 19–30 kg/m^2^; and no regular medication within the last 2 weeks prior to the first study day. Major exclusion criteria were: smoking, alcohol or drug abuse; impaired renal function with a creatinine clearance of ≤90 mL/min (calculated using the Cockroft–Gault equation); laboratory or clinical signs of any coagulation disorder; and history of seizure disorder. On the study day, eight volunteers received a single intravenous 2 g dose of cefiderocol (Fetcroja^®^) administered over 3 h using a volumetric infusion pump.

Blood samples were collected before and 1, 2, 3 (end of infusion), 3.5, 4, 5, 6, 7 and 8 h after administration of cefiderocol from each subject. At each time point, 4 mL of blood was drawn and an additional 4 mL for ultrafiltration at 3, 4 and 7 h after cefiderocol administration. The intravenous catheters were rinsed with physiological saline (0.9%) solution after sampling. Blood samples were centrifuged within 1 h at +4°C and 2600 g for 10 min, and plasma was divided into two aliquots of 1 mL and then frozen at −20°C. At the end of the study day, plasma samples were transferred to the −80°C freezer and stored until further analysis.

Free cefiderocol concentrations in subcutaneous adipose and skeletal muscle tissues were determined by MD. The MD catheter consists of a semi-permeable membrane that can be placed in the tissue of interest.^15^ Due to the low molecular cut-off of the membrane, only unbound drug can permeate the membrane and can then be collected in the MD vials. Once the probe is implanted into the tissue, substances present in the extracellular fluid at a concentration C_tissue_ are sampled into the probe and the concentration in the dialysate (C_dialysate_) can be measured. MD catheters with a molecular weight cut-off of 20 kDa and a membrane length of 10 mm (63 MD catheter, M Dialysis, Solna, Sweden) were used here. Healthy volunteers received two MD catheters in the same thigh (one in subcutaneous adipose tissue and one in muscle tissue). Microdialysate samples were obtained at baseline and during the following time intervals from each subject: 0–1, 1–2, 2–3, 3–3.5, 3.5–4, 4–5, 5–6, 6–7 and 7–8 h after the start of drug administration. The MD catheters were constantly perfused with 0.9% saline solution at a flow rate of 0.5 µL/min employing precision pumps (107 MD pump; M Dialysis AB, Stockholm, Sweden). Within 1 h of collection, MD samples were snap-frozen at approximately −20°C without further processing. At the end of the study day, MD samples were transferred from −20°C to −80°C and stored until further analysis.

For most analytes, equilibrium between extracellular tissue fluid and the perfusion medium is incomplete; therefore the concentration in tissue is higher than the concentration in dialysate. The factor by which the concentrations are interrelated is termed relative recovery (RR) and is determined by *in vivo* calibration. *In vivo* calibration was performed using the retrodialysis^16^ method at the end of the study day^17^ (two samples per healthy volunteer). The principle of this method relies on the fact that the exchange process is quantitatively equal in both directions through the semi-permeable membrane of the MD catheter. The *in vivo* RR was calculated as:


$$\mathrm{RR}\;(\text{\%})=100-\;\left(100\times \frac{\mathrm{analyte}\;\;\mathrm{concentratio}n_{\mathrm{out}}}{\mathrm{analyte}\;\;\mathrm{concentratio}n_{\mathrm{in}}}\right).$$

Interstitial concentrations were calculated according to the following equation:


$$\mathrm{Interstitial}\;\mathrm{concentrations}=100\times \frac{\mathrm{sample}\;\;\mathrm{concentration}}{in\;vivo\;\;\mathrm{RR}\;(\text{\%})}.$$

Catheters were perfused with cefiderocol at a flow rate of 0.5 µL/min for 90 min. The perfusion medium for calibration contained 75 µg/mL cefiderocol, which was chosen based on the *in vitro* experiments (Table S2). MD probes were removed at the end of the study.

### Sample analysis

Cefiderocol concentrations in plasma and microdialysate were analysed using high-performance liquid chromatography with ultraviolet detection (HPLC-UV). The HPLC consisted of a Shimadzu Prominence modular system with a degasser (DGU 20A3R), quaternary solvent pump (LC 20AD), autosampler (SIL 20AC HT, set to 6°C), column oven (CTO 20AC, set to 40°C), photodiode array detector (SPD M30A, detection wavelength 285 nm) equipped with cells of 10 mm (for plasma) or 85 mm optical path length (for microdialysate), system controller CBM 20A and LabSolution software for integration (all from Shimadzu Europe, Duisburg, Germany). The HPLC system utilized in the experiment comprised a modular Shimadzu Prominence setup that included a degasser (DGU 20A3R), a quaternary solvent pump (LC 20AD), an autosampler (SIL 20AC HT, maintained at 6°C), a column oven (CTO 20AC, set to 40°C), a system controller (CBM 20A), the LabSolution software for integration and a photodiode array detector (SPD M30A, with a detection wavelength of 285 nm). The detector was equipped with cells having either a 10 mm optical path length (for plasma) or an 85 mm optical path length (for microdialysate). Separation was performed using a Cortecs T3 2.7 µm 100 × 3 mm analytical column (Waters, Eschborn, Germany) preceded by a guard column (Nucleoshell RP18 2.7µ 4 × 3 mm column protection system, Macherey-Nagel, Düren, Germany). The mobile phase was 0.1 M sodium phosphate buffer/acetonitrile, pH 3.0, 88:12 (v/v). At a flow rate of 0.4 mL/min, cefiderocol eluted after ca. 3.5 min.

Total plasma concentrations of cefiderocol were determined according to a previously published protocol.^18^ In short, a mixture was prepared by combining 100 µL of plasma with 200 µL of 25 mM sodium dihydrogenphosphate and 500 µL of acetonitrile. An aliquot of the aqueous layer was injected into the HPLC system, after separation of the precipitated proteins and extraction of acetonitrile into dichloromethane (1.3 mL).

Free plasma concentrations of cefiderocol were determined after ultraﬁltration (of 3, 4 and 7 h plasma samples) as previously described.^19^ In brief, 10 µL of 2 M HEPES buffer (pH 7.4) was pipetted into the ultrafiltration device (Vivafree^™^ 500 30 kD Hydrosart^®^ centrifugal ultrafiltration device, Vivaproducts Inc., Littleton, MA, USA) and mixed with 300 µL plasma. The sample was incubated for 10 min at 100×g at 37°C (centrifuge 5417R; Eppendorf, Hamburg, Germany) and then centrifuged for 20 min at 1000×g at 37°C. An aliquot of the ultrafiltrate was injected into the HPLC. Microdialysate was injected directly. The injection volume was 1 µL for all matrices.

The validation data are listed in Table S1. The linearity was shown from 300–1 mg/L (R > 0.998) in plasma and from 300–0.1 mg/L (R > 0.999) in saline as surrogate for microdialysate or ultrafiltrate. The lowest non-zero calibrator concentration was used as practical lower limit of quantification (LLOQ; plasma 1 mg/L, microdialysate 0.1 mg/L). Based on back calculation of calibrator samples as well as on in-process quality controls (QCs) in plasma/saline, the coefficient of variation (CV) of intra- and inter-assay precision was <4%/<3% and the relative error in accuracy was <5%/<5%. The unbound fraction (fu = free concentration/total concentration × 100%) of cefiderocol in QCs (concentrations 50 and 10 mg/L) was 67.4 ± 2.1%, corresponding to an inter-assay precision of 3.1% (CV). Regarding free cefiderocol plasma concentrations, accuracy cannot be determined since protein binding in the individual plasma samples is not known.^20^ The processed samples were sufficiently stable overnight in the autosampler at 6°C (solution for total plasma concentration measurement 99.5%, plasma ultrafiltrate 96.9%, microdialysate 92.7%).

### Non-compartmental analysis (NCA)

The PK outcome variables area under the curve after 8 h (AUC_0-8h_), maximum recorded concentration (C_max_), time it takes to reach C_max_ (T_max_), volume of distribution (Vd), clearance (CL) and half-life (t_1/2_) were determined for the different compartments, if applicable (plasma, subcutaneous and muscle tissue). Plasma PK parameters were calculated with total and unbound concentrations. Unbound plasma concentrations were calculated using the mean plasma protein binding (PPB) of each individual subject. The RR of each individual subject was used to calculate the concentration at the MD insertion site. The non-compartmental analysis (NCA) was performed using Phoenix WinNonLin (Certara, USA).

### Population PK analysis

A population PK model describing cefiderocol PK in plasma, subcutaneous adipose and muscle tissue was developed. One- and two-compartment models with first-order elimination were evaluated to describe plasma concentrations. Estimates were based on unbound cefiderocol concentrations. The unbound fraction was estimated based on the difference between total and free cefiderocol concentrations as determined by ultrafiltration (taken after 3, 4 and 7 h after cefiderocol infusion start). When modelling tissue PK, the parameter estimates describing plasma PK were initially fixed, while all parameters were simultaneously estimated in the final model. Microdialysate data were corrected using the RR value determined for each individual catheter. The recovery-corrected MD data were modelled using an established approach integrating the tissue concentration–time curve in each collection interval.^21^ Models with separate tissue compartments with or without mass transfer from and to the central plasma compartment were evaluated, as well as models in which tissue concentrations were scaled to concentrations in the central or peripheral compartment of the plasma PK model.

Inter-individual variability (IIV) was evaluated for all structural model parameters and included using an exponential model. Residual unexplained variability was evaluated using proportional, additive, and combined models for plasma and tissue data. The small and homogeneous healthy volunteer population precluded a systematic covariate analysis. Model evaluation and discrimination were based on the objective function value (ΔOFV > 3.84 for nested models with 1 df, α = 0.05), goodness-of-fit plots, precision and plausibility of parameter estimates and visual predictive checks (VPCs; *n* = 1000).^22^ Nonlinear mixed-effects modelling was performed using NONMEM 7.4 (first-order conditional estimation with interaction)^23^ assisted by PsN 5.3.0,^24^ and Pirana (v21.11.1).^25^ Visualization of data and statistical analyses were carried out in R 4.2.2.^26^

### PTA analysis

Monte Carlo simulations (*n* = 5000 subjects) were performed using the final population PK model to determine the PTA. PTA was calculated based on simulations of the first 24 h of a 2 g q8h dosing regimen (IV over 3 h), as recommended for adult patients without renal impairment. The PK/PD targets *ƒ*T_>MIC _= 75% and *ƒ*T_>MIC _= 95% (of the dosing interval) were used. *ƒ*T_>MIC _= 75% had been associated with a 1 − log_10_ reduction in bacterial count against Enterobacteriaceae and *Pseudomonas aeruginosa* in a neutropenic murine thigh infection model.^27^ Achieving a target of 100% fT_>MIC_ is impossible, since the PTA is based on the first 24 h of the treatment. Therefore, 95% fT_>MIC_ was used as a substitute for 100% fT_>MIC_, which is commonly used as a PK/PD target for beta-lactam antibiotics.

## Results

### *In vitro* experiments

The mean recovery and loss rates for the MD probes at each time interval are shown in Table S2. The mean value (±standard deviation) for recovery during forward dialysis was 37.7% (±3.4%). The mean value (±standard deviation) for loss during retrodialysis was 35.8% (±1.7%). The recovery and loss values showed consistency across the different sampling intervals and in both directions (retrodialysis and forward dialysis). Since recovery is usually much lower in the *in vivo* setting, we decided to reduce the flow rate for the *in vivo* setting to 0.5 µg/L for both MD probes.

### Study population and safety

The average age and body mass index of the eight participants were 34.9 ± 8.9 years and 24.6 ± 1.1 kg/m^2^, respectively. Cefiderocol was generally well tolerated. Three participants experienced headache and another volunteer experienced heartburn. All adverse effects were classified as not related to the study medication by the investigator, were self-limiting and did not pose a safety risk. None of the commonly reported adverse reactions of cefiderocol (diarrhoea, vomiting, nausea and cough^9^) were observed in the healthy volunteers in the present study.

### Pharmacokinetic parameters calculated with NCA

**Figure 1.**
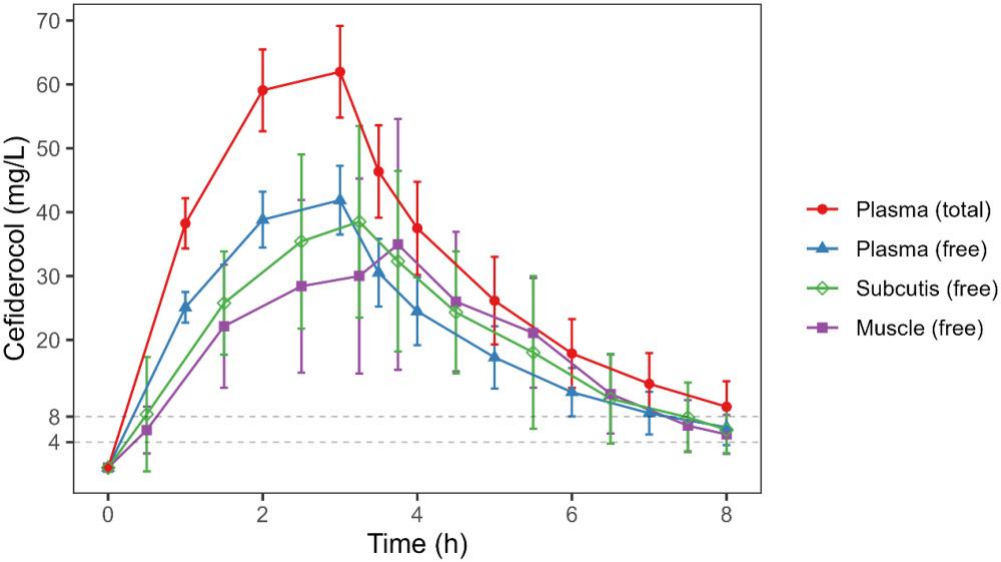
Concentration–time profile of cefiderocol after a single intravenous dose of 2 g (mean ± SD). Tissue concentrations are plotted at the mid-point of the microdialysate collection interval. The dotted horizontal lines indicate an MIC of 4 and 8 mg/L. This figure appears in colour in the online version of *JAC* and in black and white in the print version of *JAC*.

The mean and individual concentration–time profiles based on free and total drug concentrations in plasma, subcutaneous adipose and skeletal muscle tissue are shown in Figure 1 and Figure S2. Two microdialysate observations of the subcutaneous adipose tissue were not included in the NCA analysis because of their implausibility. Cefiderocol PK parameters calculated with NCA for plasma, muscle and subcutaneous tissue are shown in Table 1.

**Table 1.** Pharmacokinetic parameters of cefiderocol after a single intravenous dose of 2 g administered over 3 h given as mean ± standard deviation

Compartment	t_½_ (h)	C_max_ (mg/L)	AUC_0-8h_ (h ∗ mg/L)	Vd (L)	CL (L/h)	Penetration ratio
Subcutis (free)	1.82 ± 0.43	41.6 ± 10.5	163.2 ± 61.9	NA	NA	0.99 ± 0.33
Muscle (free)	1.30 ± 0.41	40.9 ± 14.5	151.7 ± 58.2	NA	NA	0.92 ± 0.30
Plasma (free)	2.04 ± 0.41	39.9 ± 5.43	162.0 ± 24.3	32.7 ± 4.81	11.4 ± 1.94	

AUC, area under the concentration–time curve; CL, total body clearance; C_max_, maximum concentration; NA, not applicable; MIC, minimum inhibitory concentration; T_max_, time to maximum concentration; t_½_ , terminal elimination half-life; Vd, volume of distribution.

C_max_ of free cefiderocol in plasma, muscle tissue and subcutaneous tissue were comparable (39.9 ± 5.43 mg/L versus 40.9 ± 14.5 mg/L versus 41.6 ± 10.5 mg/L). The mean fraction unbound was 66.0 ± 3.47% and was independent of the observed concentration. The AUC_0-8h_ values were comparable for plasma, subcutis and muscle. Penetration ratios (AUC_0-8h_free_tissue_/AUC_0-8h_free_plasma_) were close to 1 for both subcutis and muscle (Table 1).

### Population PK model and PTA analysis

**Figure 2.**
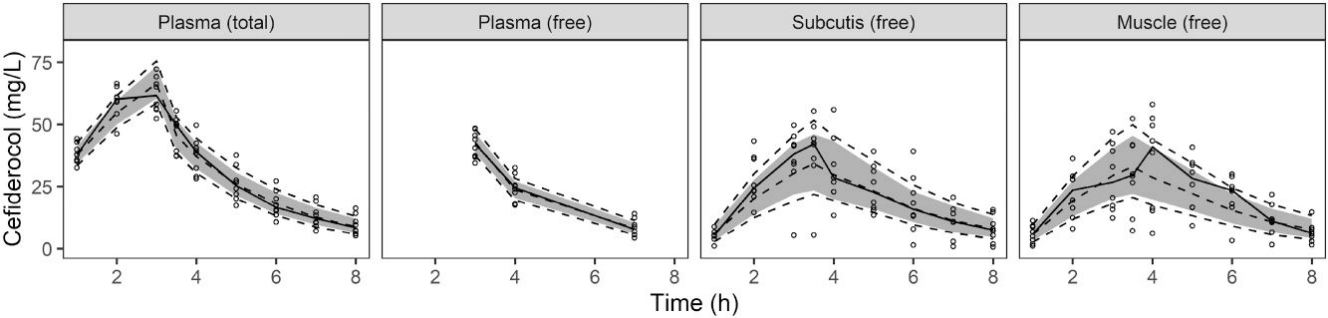
Visual predictive check for the population pharmacokinetic model. Open circles represent observations, and the solid line indicate the median of observed concentrations. The dashed lines represent the 10th, 50th and 90th percentiles of the simulated data (*n* = 1000). The shaded areas indicate the 95% confidence interval around the simulated median. Note that the confidence intervals around the 10th and 90th percentiles of the simulated data are not plotted given the small population size.

A two-compartment model with IIV on CL and Vc and a proportional error model best described the plasma PK of cefiderocol (Table 2). Interstitial tissue concentrations were described by scaling the tissue concentrations to concentrations in the peripheral compartment of the plasma model. A model with separate compartments for the tissue data resulted in stability/estimation issues. The residual unexplained variability for tissue PK was described using one combined error model, since separate models for each tissue were associated with low precision and comparable parameter estimates but no significantly better model fit. Two microdialysate observations of the subcutaneous adipose tissue were considered implausible given the individual concentration–time profiles and were not considered in the model due to a disproportionate influence on model fit, parameter estimates and model stability. Goodness-of-fit plots and VPCs (Figure 2 and Figure S3) indicated overall adequate model performance. Median concentration–time profiles, which should be interpreted cautiously due to the small sample size of only eight individuals, indicated that the model did not optimally capture a potential delay in distribution to the muscle tissue; a model with additional transit compartments, however, was not supported by the data.

**Table 2.** Parameter estimates of the population pharmacokinetic model

Parameter	Description	Estimate (RSE%)
Fixed effects		
CL (L/h)	Clearance	11.0 (6.7)
V_c_ (L)	Distribution volume central compartment	11.3 (16.3)
f_u_	Fraction unbound in plasma	0.639 (1.5)
Q_12_ (L/h)	Inter-compartmental clearance	25.3 (23.0)
V_p_ (L)	Distribution volume peripheral compartment	15.1 (14.2)
TF_sub_	Scaling factor subcutaneous adipose tissue	0.943 (10.5)
TF_mus_	Scaling factor muscle tissue	0.901 (11.2)
Inter-individual variability		
CL (CV%)		17.4 (15.4)
V_c_ (CV%)		26.5 (17.1)
TF_sub_ (CV%)		28.0 (21.3)
TF_mus_ (CV%)		25.2 (35.8)
Residual error		
Prop_plasma_ (CV%)	Proportional error plasma PK	5.85 (6.9)
Prop_tissue_ (CV%)	Proportional error tissue PK	30.3 (13.5)
Add_tissue_ (mg/L)	Additive error tissue PK	2.3 (5.6)

Estimates are based on unbound cefiderocol concentrations.

RSE, relative standard error; CV%, coefficient of variation, calculated according to $\sqrt{e^{\omega^{2}}-1}x\times 100\text{\%}$.

**Figure 3.**
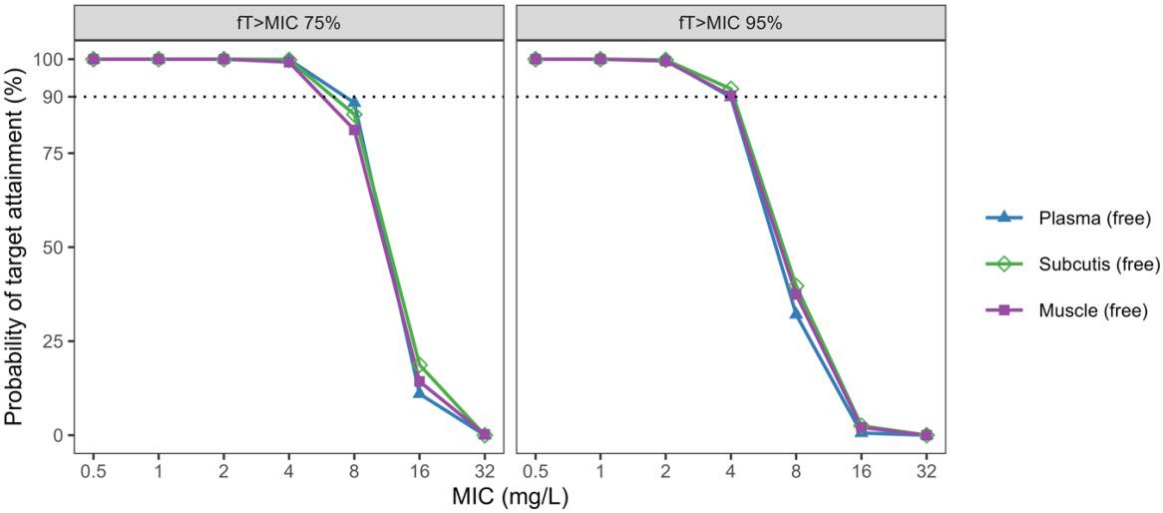
Probability of target attainment (PTA) based on cefiderocol pharmacokinetics in plasma, subcutaneous adipose tissue and skeletal muscle tissue over 24 h following a 2 g q8h dosing regimen given as an intravenous infusion over 3 h. PTA was calculated using a *f*T_>MIC_ target of 75% and 95%. The dotted line indicates 90% PTA. This figure appears in colour in the online version of *JAC* and in black and white in the print version of *JAC*.

PTA was assessed based on the first 24 h of treatment using the PK/PD target ƒT > MIC = 75% and ƒT > MIC = 95%. For the cefiderocol dosing regimen recommended in patients without renal impairment (2 g q8h as intravenous infusion over 3 h), PTA was ≥90% for MIC values up to 4 mg/L, both based on free plasma and tissue PK (Figure 3).

## Discussion

The present study determined the PK and target attainment of cefiderocol in subcutaneous adipose, muscle tissue and plasma of eight healthy volunteers. The total plasma PK parameters determined with NCA are in agreement with two previous studies: we report a Vd of 21.2 L (which was 20.9 L in Saisho *et al*.^12^), t_1/2_ of 2.1 h (which was 2.5–3 h in Saisho *et al*.^12^ and 2.3 h in Miyazaki *et al*.^28^) and a CL of 7.2 L/h (compared to 5.1^12^ and 4.8 L/h^28^).

Cefiderocol penetrates rapidly and extensively into subcutaneous adipose and skeletal muscle tissue, with unbound AUC ratios of 0.94 and 0.89, respectively. The relatively high molecular weight of cefiderocol appears not to considerably affect its distribution into soft tissues.

We report an average cefiderocol PPB of 34% (*n* = 8 subjects, three samples per subject), which differs from the previously reported value of 58% reported by Matsumoto *et al.*^29^ and 40%–60% reported by the manufacturer^9^; unfortunately, sample sizes and the degree of variability are not provided. Katsube *et al*.^30^ reported similar values (about 60% PPB) in subjects with various degrees of renal impairment (*n* = 30) and in healthy subjects (*n* = 8), with coefficients of variation ranging from 9.8% to 43.5%. While differences in the study population might explain some variation, the markedly lower PPB as found in the present study is more likely caused by methodological issues. In the present study, free concentrations were determined by a validated ultrafiltration method.^19^ Factors favouring a higher PPB were low temperature and high centrifugal forces during ultrafiltration. Considering the limited stability of cefiderocol in (unbuffered) serum, also thermal stress can lead to lower free concentrations, resulting in a seemingly higher PPB.^31^ However, even if previously reported protein binding data may be imprecise (considering the high variability), the difference in the mean values between the previous and present results remains unexplained and needs further investigations. Nevertheless, the high tissue penetration of cefiderocol as found in the present study is in good agreement with a low PBB of cefiderocol.

The commonly used PTA threshold of 90% was attained for MIC values up to 4 mg/L, based on simulations of plasma PK as well as soft tissue PK for both PK/PD targets. The results of the plasma PTA analysis are in line with a previously published study that used the PK/PD target of *ƒ*T_>MIC _= 75%.^32^ Based on plasma concentration data of infected patients, the authors of this study developed a population PK model and performed a PTA analysis. Similar to the present study, for an intravenous cefiderocol dose of 2 g every 8 h, the PTA was ≥90% for MICs of ≤4 mg/L. Notably, for an MIC of 8 mg/L and the target of *ƒ*T_>MIC _= 75%, the plasma PTA was just below 90% and the tissue PTA between 75% and 90%. For the target of ƒT_>MIC _= 95% and an MIC of 8 mg/L, the plasma and tissue PTA was below 50%.

To understand the PTA results in a clinical and microbiological context, it’s important to consider the MIC of the specific pathogens that cefiderocol is intended to target. According to the European Medicines Agency, cefiderocol should be used only against aerobic Gram-negative organisms in patients with limited treatment options. Wang *et al*.^33^ reviewed the *in vitro* activity data of cefiderocol collected from 38 studies that included 34 805 Enterobacterales and 8297 *P. aeruginosa* isolates. The MIC_90_ for Enterobacterales ranged between 0.5 and 4 mg/L in the majority of studies. For *P. aeruginosa*, the MIC_90_ was always ≤1 mg/L. Yet, cefiderocol is predominantly used against MDR isolates in cases where few or no other treatment options are available. The MIC_90_ of cefiderocol was reported as 1 mg/L against meropenem-resistant *P. aeruginosa* and 8 mg/L against ceftolozane–tazobactam-resistant *P. aeruginosa.* Against meropenem-resistant *Acinetobacter* spp., the MIC_90_ of cefiderocol was reported as 2–4 mg/L. Most of the resistant phenotypes of Enterobacterales showed an MIC_90_ ≤ 4 mg/L against cefiderocol, but the MIC_90_ against ceftazidime–avibactam-resistant isolates was 8 mg/L.^34,35^

Taking together the favourable target attainment up to an MIC of 4 mg/L in plasma and the reported MIC_90_ values, good efficacy can be expected against most of the relevant pathogens. For tissue, a PK/PD target has not been established but assuming a similar PK/PD target as for plasma, the PTA analysis supports the use of cefiderocol for the treatment of SSTIs. Of note, the relevant PTA threshold (90%) was not reached for an MIC of 8 mg/L corresponding to the MIC_90_ of ceftazidime–avibactam-resistant Enterobacterales and ceftolozane–tazobactam-resistant *P. aeruginosa*.^35^ This finding indicates that only a suboptimal antimicrobial effect can be achieved in these cases. Further studies assessing the plasma and tissue PK in patients at risk of developing MDR-GNB infections, such as critically ill, burn and diabetic patients, are needed to confirm the present findings.

The current study has some limitations. Only a limited number of healthy volunteers were included, and the results may thus not reflect the PK variability in patient populations. The infection pathophysiology can alter drug PK, resulting in inadequate drug exposure.^36^ Moreover, this study was performed in only eight healthy subjects. Therefore, extrapolation of the study results to patients should be exercised with caution. Finally, the cefiderocol PK/PD target is based on plasma PK data, and may not apply to tissue PK/PD.^37,38^

### Conclusion

The present study demonstrated that an intravenous infusion of 2 g cefiderocol achieves sufficiently high concentrations for the treatment of the most relevant bacterial species in plasma and soft tissues. These findings support the use of cefiderocol for SSTIs caused by MDR-GNB. Further investigations in critically ill patients, burn patients and diabetic patients are needed to confirm these findings.

## Supplementary Material

dkae359_Supplementary_Data

## Supplementary data

Figures S1–S3 and Tables S1 and S2 are available as Supplementary data at *JAC* Online.

## References

[1] Antimicrobial Resistance Collaborators . Global burden of bacterial antimicrobial resistance in 2019: a systematic analysis. Lancet 2022; 399: 629–55. 10.1016/S0140-6736(21)02724-0 35065702 PMC8841637

[2] Jabbour J-F , Sharara SL, Kanj SS. Treatment of multidrug-resistant Gram-negative skin and soft tissue infections. Curr Opin Infect Dis 2020; 33: 146–54. 10.1097/QCO.0000000000000635 32022742

[3] Russo A , Trecarichi EM, Torti C. The role of Gram-negative bacteria in skin and soft tissue infections. Curr Opin Infect Dis 2022; 35: 95–102. 10.1097/QCO.0000000000000807 34840273

[4] Russo A , Vena A, Bassetti M. Antibiotic treatment of acute bacterial skin and skin structure infections. Curr Opin Infect Dis 2022; 35: 120–7. 10.1097/QCO.0000000000000822 35245247

[5] Wu JY , Srinivas P, Pogue JM. Cefiderocol: a novel agent for the management of multidrug-resistant Gram-negative organisms. Infect Dis Ther 2020; 9: 17–40. 10.1007/s40121-020-00286-6 32072491 PMC7054475

[6] Ito A , Sato T, Ota M et al In vitro antibacterial properties of cefiderocol, a novel siderophore cephalosporin, against Gram-negative bacteria. Antimicrob Agents Chemother 2018; 62: e01454-17. 10.1128/AAC.01454-17 PMC5740388 29061741

[7] Bassetti M , Echols R, Matsunaga Y et al Efficacy and safety of cefiderocol or best available therapy for the treatment of serious infections caused by carbapenem-resistant Gram-negative bacteria (CREDIBLE-CR): a randomised, open-label, multicentre, pathogen-focused, descriptive, phase 3 trial. Lancet Infect Dis 2021; 21: 226–40. 10.1016/S1473-3099(20)30796-9 33058795

[8] Wunderink RG , Matsunaga Y, Ariyasu M et al Cefiderocol versus high-dose, extended-infusion meropenem for the treatment of Gram-negative nosocomial pneumonia (APEKS-NP): a randomised, double-blind, phase 3, non-inferiority trial. Lancet Infect Dis 2021; 21: 213–25. 10.1016/S1473-3099(20)30731-3 33058798

[9] SmPC . Fetcroja 1g Powder for concentrate for solution for infusion SmPC - EMA.

[10] FDA . Fetroja 1g Powder for concentrate for solution for infusion SmPC - FDA.

[11] Bilal M , El Tabei L, Büsker S et al Clinical pharmacokinetics and pharmacodynamics of cefiderocol. Clin Pharmacokinet 2021; 60: 1495–508. 10.1007/s40262-021-01063-5 34420182 PMC8613110

[12] Saisho Y , Katsube T, White S et al Pharmacokinetics, safety, and tolerability of cefiderocol, a novel siderophore cephalosporin for Gram-negative bacteria, in healthy subjects. Antimicrob Agents Chemother 2018; 62: e02163-17. 10.1128/AAC.02163-17 29311072 PMC5826143

[13] Sanabria C , Migoya E, Mason JW et al Effect of cefiderocol, a siderophore cephalosporin, on QT/QTc interval in healthy adult subjects. Clin Ther 2019; 41: 1724–1736.e4. 10.1016/j.clinthera.2019.07.006 31378318

[14] MacVane SH , Housman ST, Nicolau DP. In vitro microdialysis membrane efficiency of broad-spectrum antibiotics in combination and alone. Clin Pharmacol 2014; 6: 97–101. 10.2147/CPAA.S65389 24940084 PMC4051625

[15] Schmidt S , Banks R, Kumar V et al Clinical microdialysis in skin and soft tissues: an update. J Clin Pharmacol 2008; 48: 351–64. 10.1177/0091270007312152 18285620

[16] Stenken JA . Methods and issues in microdialysis calibration. Anal Chim Acta 1999; 379: 337–58. 10.1016/S0003-2670(98)00598-4

[17] Melgaard L , Hersini KJ, Gazerani P et al Retrodialysis: a review of experimental and clinical applications of reverse microdialysis in the skin. Skin Pharmacol Physiol 2013; 26: 160–74. 10.1159/000351341 23751503

[18] Kratzer A , Schießer S, Matzneller P et al Determination of total and free ceftolozane and tazobactam in human plasma and interstitial fluid by HPLC-UV. J Pharm Biomed Anal 2019; 163: 34–8. 10.1016/j.jpba.2018.09.044 30278324

[19] Lier C , Dejaco A, Kratzer A et al Free serum concentrations of antibiotics determined by ultrafiltration: extensive evaluation of experimental variables. Bioanalysis 2024; 16: 747–56. 10.1080/17576180.2024.2365526 39041640 PMC11389746

[20] Nilsson LB . The bioanalytical challenge of determining unbound concentration and protein binding for drugs. Bioanalysis 2013; 5: 3033–50. 10.4155/bio.13.274 24320129

[21] Tunblad K , Hammarlund-Udenaes M, Jonsson EN. An integrated model for the analysis of pharmacokinetic data from microdialysis experiments. Pharm Res 2004; 21: 1698–707. 10.1023/B:PHAM.0000041468.00587.c6 15497699

[22] Nguyen THT , Mouksassi M-S, Holford N et al Model evaluation of continuous data pharmacometric models: metrics and graphics. CPT Pharmacometrics Syst Pharmacol 2017; 6: 87–109. 10.1002/psp4.12161 27884052 PMC5321813

[23] Boeckmann AJ , Sheiner LB, Beal SL et al NONMEM Users Guide. NONMEM Project Group University of California at San Francisco, 2011.

[24] Lindbom L , Pihlgren P, Jonsson N. PsN-Toolkit—a collection of computer intensive statistical methods for non-linear mixed effect modeling using NONMEM. Comput Methods Programs Biomed 2005; 79: 241–57. 10.1016/j.cmpb.2005.04.005 16023764

[25] Keizer RJ , van Benten M, Beijnen JH et al Piraña and PCluster: a modeling environment and cluster infrastructure for NONMEM. Comput Methods Programs Biomed 2011; 101: 72–9. 10.1016/j.cmpb.2010.04.018 20627442

[26] R Core Team . R: A Language and Environment for Statistical Computing. R Foundation for Statistical Computing, 2021.

[27] Nakamura R , Ito-Horiyama T, Takemura M et al In vivo pharmacodynamic study of cefiderocol, a novel parenteral siderophore cephalosporin, in murine thigh and lung infection models. Antimicrob Agents Chemother 2019; 63: e02031-18. 10.1128/AAC.02031-18 31262762 PMC6709502

[28] Miyazaki S , Katsube T, Shen H et al Metabolism, excretion, and pharmacokinetics of [^14^C]-cefiderocol (S-649266), a siderophore cephalosporin, in healthy subjects following intravenous administration. J Clin Pharmacol 2019; 59: 958–67. 10.1002/jcph.1386 30730562 PMC6593826

[29] Matsumoto S , Singley CM, Hoover J et al Efficacy of cefiderocol against carbapenem-resistant Gram-negative bacilli in immunocompetent-rat respiratory tract infection models recreating human plasma pharmacokinetics. Antimicrob Agents Chemother 2017; 61: e00700-17. 10.1128/AAC.00700-17 28630178 PMC5571323

[30] Katsube T , Echols R, Arjona Ferreira JC et al Cefiderocol, a siderophore cephalosporin for Gram-negative bacterial infections: pharmacokinetics and safety in subjects with renal impairment. J Clin Pharmacol 2017; 57: 584–91. 10.1002/jcph.841 27874971 PMC5412848

[31] Zimmer J , Röhr AC, Kluge S et al Validation and application of an HPLC-UV method for routine therapeutic drug monitoring of cefiderocol. Antibiot (Basel, Switzerland) 2021; 10: 242. 10.3390/antibiotics10030242 PMC7997268 33670891

[32] Kawaguchi N , Katsube T, Echols R et al Population pharmacokinetic and pharmacokinetic/pharmacodynamic analyses of cefiderocol, a parenteral siderophore cephalosporin, in patients with pneumonia, bloodstream infection/sepsis, or complicated urinary tract infection. Antimicrob Agents Chemother 2021; 65: e01437-20. 10.1128/AAC.01437-20 33257454 PMC8092503

[33] Wang C , Yang D, Wang Y et al Cefiderocol for the treatment of multidrug-resistant Gram-negative bacteria: a systematic review of currently available evidence. Front Pharmacol 2022; 13: 896971. 10.3389/fphar.2022.896971 35496290 PMC9039133

[34] Santerre Henriksen A , Jeannot K, Oliver A et al In vitro activity of cefiderocol against European *Pseudomonas aeruginosa* and *Acinetobacter* spp., including isolates resistant to meropenem and recent β-lactam/β-lactamase inhibitor combinations. Microbiol Spectr 2024; 12: e0383623. 10.1128/spectrum.03836-23 38483164 PMC10986614

[35] Shortridge D , Streit JM, Mendes R et al In vitro activity of cefiderocol against U.S. and European Gram-negative clinical isolates collected in 2020 as part of the SENTRY antimicrobial surveillance program. Microbiol Spectr 2022; 10: e0271221. 10.1128/spectrum.02712-21 35262394 PMC9045385

[36] Sanz Codina M , Zeitlinger M. Biomarkers predicting tissue pharmacokinetics of antimicrobials in sepsis: a review. Clin Pharmacokinet 2022; 61: 593–617. 10.1007/s40262-021-01102-1 35218003 PMC9095522

[37] van Os W , Zeitlinger M. Predicting antimicrobial activity at the target site: pharmacokinetic/pharmacodynamic indices versus time-kill approaches. Antibiot (Basel, Switzerland) 2021; 10: 1485. 10.3390/antibiotics10121485 PMC8698708 34943697

[38] Bulitta JB , Hope WW, Eakin AE et al Generating robust and informative nonclinical in vitro and in vivo bacterial infection model efficacy data to support translation to humans. Antimicrob Agents Chemother 2019; 63: e02307-18. 10.1128/AAC.02307-18 30833428 PMC6496039

