# Supplementary material for: Target-site cefiderocol pharmacokinetics in soft tissues of healthy volunteers
Source: J Antimicrob Chemother. 2024 Oct 7;79(12):3281–8. doi: 10.1093/jac/dkae359 (PMC11638555; doi:10.1093/jac/dkae359)
Supplement: dkae359_Supplementary_Data [file dkae359_supplementary_data.docx]

**Supplemental Material**

**Target-Site Cefiderocol Pharmacokinetics in soft tissues of healthy volunteers**


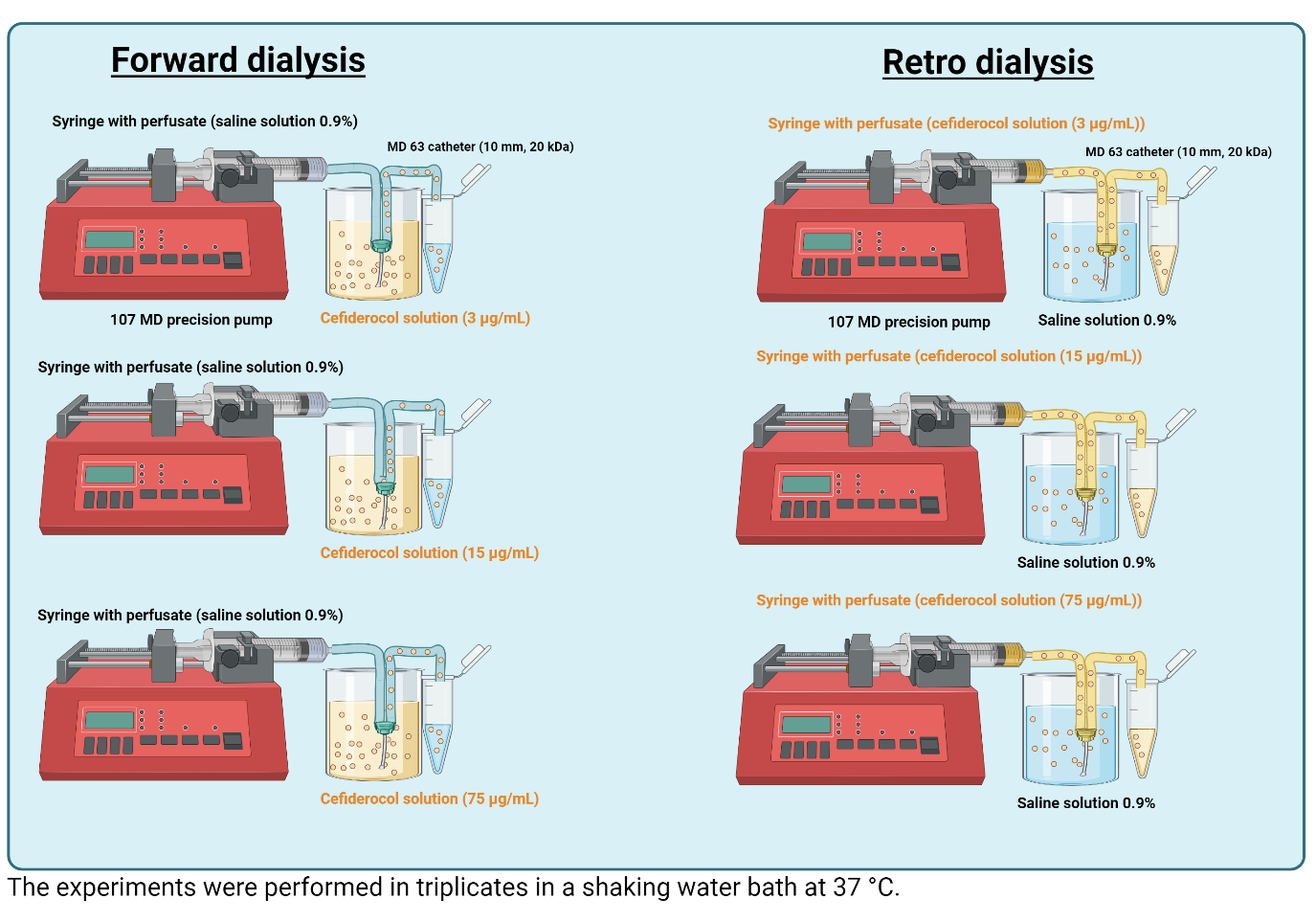


**Figure S1:** In vitro microdialysis experiments. Three MD catheters (single use MD 63 catheters, M Dialysis AB, Stockholm, Sweden) with membranes of a molecular weight cut-off of 20 kDa and a membrane length of 10 mm were used. MD Catheters were connected with precision pumps (107 MD pump; M Dialysis AB, Stockholm, Sweden). The immersion solutions were placed in 10 mL glass vials and plastic vials were used for the collection of the microdialysate.


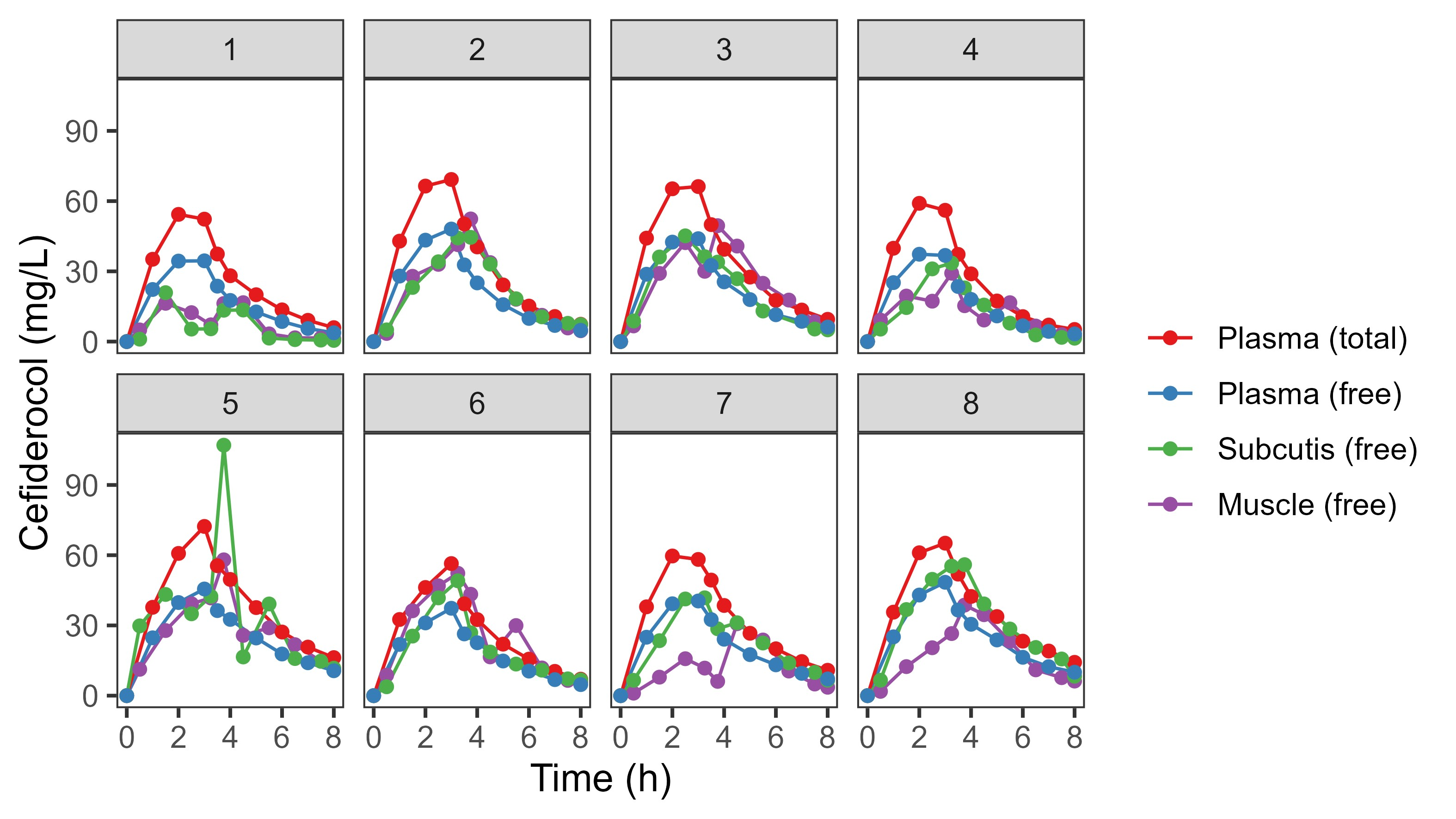


**Figure S2:** Concentration-time profiles of cefiderocol in plasma (total and unbound) and microdialysate of subcutaneous adipose and muscle tissue in eight healthy subjects following extended intravenous infusion (3 h) of 2000 mg cefiderocol.


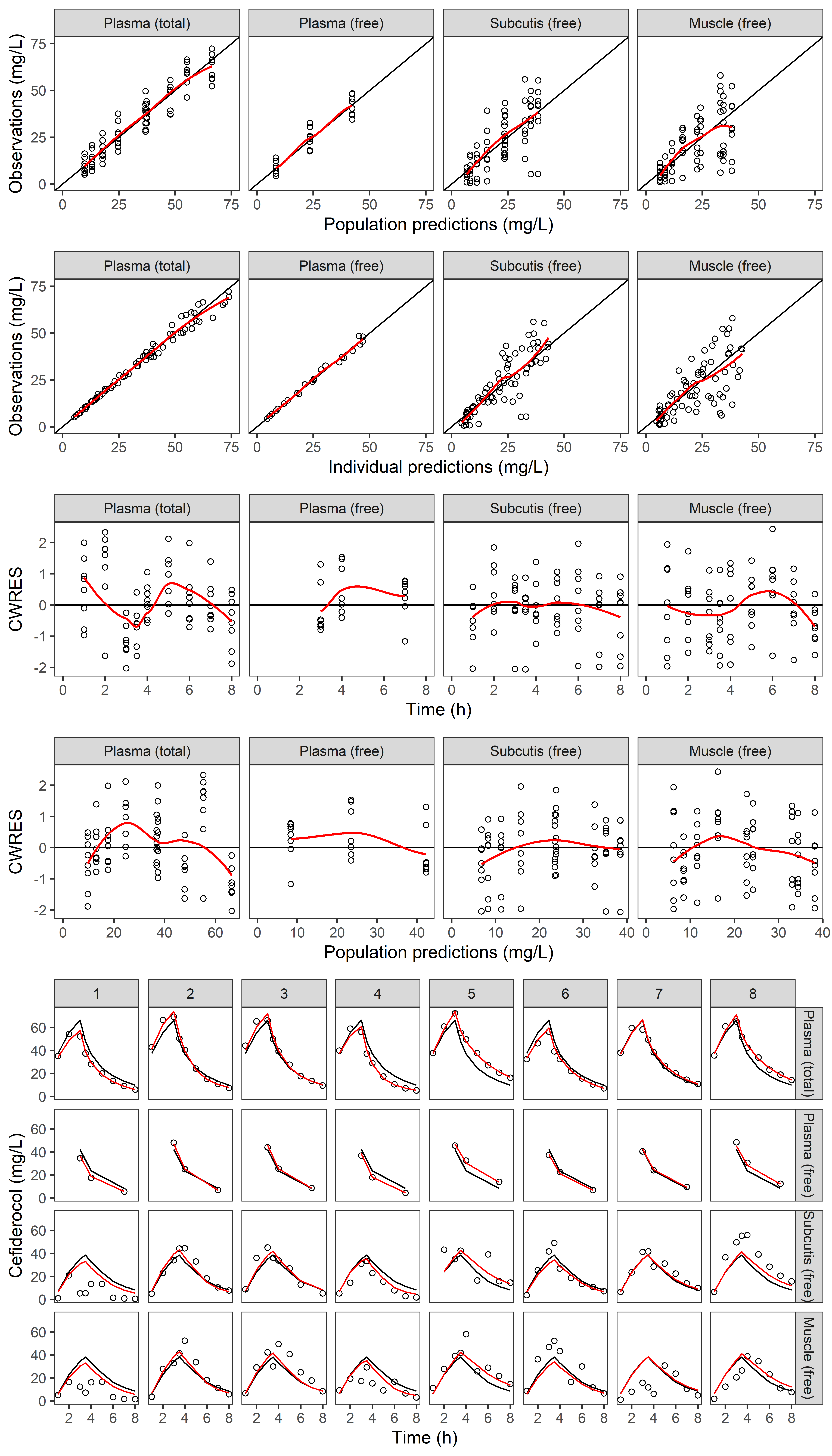


**Figure S3.** Goodness-of-fit plots for the cefiderocol population pharmacokinetic model. In the top four plots, red lines indicate Loess smoothers. In the bottom plot, red lines indicate the individual model predictions and black lines indicate the typical predictions.

CWRES: conditional weighted residuals

**Table S1**: Validation data (mean±SD or range) for determining cefiderocol in plasma or microdialysate.

|  | Plasma | Saline^1)^ |
| --- | --- | --- |
| Linearity^2)^ | 300-1 mg/L | 300-0.1 mg/L |
| R | >0.998 | >0.999 |
| LLOQ10σ^3)^ | 0.294 | 0.017 |
| LLOD^4)^ | 10 pg on-column | |
| Recovery | 87.9 ± 2.3 % | NA |
| Precison CVintra^5)^ | ND | ND |
| CVintra^5)^ | 0.71-3.1 % | 0.53-2.0 % |
| Accuracy^5)^ | 95.2-101 % | 98.3-101 % |

*1) Surrogate for ultrafiltrate or microdialysate.*

*2) Calibration according to y = B0 + B1*x, weighting factor 1/y^2^.*

*3) Estimated LLOQ according to LLOQ = 10(σ_y_/S) with σ_y_ = standard deviation of the response and S = the slope of the calibration curve.*

*4) Amount of analyte injected in a pure solvent, which caused a signal-to-noise ratio of S/N = 3*

*5) Back calculated values, including the lowest calibrator concentration.*

**Table S2:** Mean recovery/loss rates (%) of the *in vitro* microdialysis experiments with concentrations of 3, 15, and 75 µg/ml cefiderocol with physiological saline as perfusion solution with a 2 µL/min flow rate.

| **Cefiderocol concentration** | **Time** | **Forward dialysis** | **Reverse dialysis** |
| --- | --- | --- | --- |
| **3 µg/ml** | 0-0.5 h | 39.4% | 40.6% |
|  | 0.5-1 h | 39.3% | 37.8% |
|  | 1-2 h | 40.5% | 36.1% |
|  | 2-3 h | 40.1% | 36.3% |
|  |  |  |  |
| **15 µg/ml** | 0-0.5 h | 39.8% | 34.8% |
|  | 0.5-1 h | 39.7% | 35.1% |
|  | 1-2 h | 39.5% | 35.8% |
|  | 2-3 h | 38.7% | 34.8% |
|  |  |  |  |
| **75 µg/ml** | 0-0.5 h | 35.8% | 37.1% |
|  | 0.5-1 h | 34.1% | 34.6% |
|  | 1-2 h | 32.9% | 33.4% |
|  | 2-3 h | 32.2% | 33.2% |
| **Mean±SD** |  | **37.7% ± 3.40%** | **35.8% ± 1.67%** |
